# Inflatable penile prosthesis as tissue expander: what is the evidence?

**DOI:** 10.1590/S1677-5538.IBJU.2016.0528

**Published:** 2017

**Authors:** Paul H. Chung, Jordan A. Siegel, Timothy J. Tausch, Alexandra K. Klein, Jeremy M. Scott, Allen F. Morey

**Affiliations:** 1 Department of Urology, University of Texas Southwestern Medical Center, Dallas, TX, USA

**Keywords:** Penile Prosthesis, Erectile Dysfunction, Surgical Procedures, Operative

## Abstract

**Objective:**

Many patients who undergo inflatable penile prosthesis (IPP) replacement are often upsized to larger cylinders, suggesting the IPP may serve as a tissue expander and increase internal penile length. The objective of this study is to evaluate whether cylinder length increases with subsequent IPP insertion.

**Materials and Methods:**

We queried American Medical Systems and Coloplast Patient Information Form databases to identify patients who underwent IPP placement and replacement between 2004-2013. Patients were grouped by device type and time to replacement (<2 or ≥2 years). We selected the 2-year mark for subgroup analysis to allow time for tissue expansion to occur and to exclude patients who underwent early explantation (e.g. erosion or infection).

**Results:**

Two thousand, seven hundred and forty nine patients (1,532 AMS 700 LGX, 717 AMS 700 CX, and 500 Coloplast Titan) met the inclusion criteria. Mean time between implants was earlier for LGX (29 months) than CX (39 months) and Titan (48 months) patients (p<0.001). Patients who underwent device replacement at <2 years did not experience an increase in mean cylinder length. On the contrary, patients who underwent device replacement at ≥2 years did experience significant increases in mean cylinder length (LGX 1.2 cm, CX 1.1 cm, and Titan 0.9 cm, p<0.001). The mean increases in length at ≥2 years were similar between the 3 devices (p=0.20). Sixty percent of patients demonstrated increases of >0.5 cm and 40% demonstrated increases of ≥1 cm.

**Conclusions:**

As demonstrated, the IPP may provide tissue expansion over time. Further evaluation is needed to determine if increased cylinder length correlates to increased functional length and patient satisfaction.

## INTRODUCTION

Although patients report high rates of satisfaction with penile prosthesis surgery, penile shortening has long been recognized as a common patient complaint (1–6).

Patients often attribute penile shortening to the prosthesis; however, many patients fail to acknowledge that radical prostatectomy, Peyronie's disease, priapism, long-standing erectile dysfunction, and obesity can all affect penile length even prior to device placement (7). The largest study to evaluate post-operative penile length showed that prosthesis do not shorten post-operative stretched penile length (5). In addition, another study suggested that the combination of aggressive sizing with a penile rehabilitation inflation protocol may actually help to increase post-operative stretched penile length (4).

We have often observed that patients are often upsized to larger cylinders during inflatable penile prosthesis (IPP) replacement surgery. We hypothesized that IPP may serve as tissue expanders, stretching the corpora gradually over time when inflated regularly and increase internal penile length. We used IPP cylinder length as a surrogate for internal penile length and queried industry data to evaluate on a nationwide scale whether IPP cylinder length increases at the time of device replacement.

## MATERIALS AND METHODS

Study requests were submitted to and approved by both American Medical Systems (AMS, Minneapolis, MN, USA) and Coloplast (Minneapolis, MN, USA) to obtain Patient Information Form (PIF) data for patients who had IPP placement and replacement (AMS 700LGX, AMS 700CX, and Coloplast Titan) between 2004 and 2013. Cylinder lengths were calculated as the total length of both cylinders plus rear tip extenders from each side. The average length from both sides was used for patients with mismatched lengths. Differences in IPP length were stratified by device type and by the interval duration between surgical dates of device replacement (<2 or ≥2 years); reason for device replacement was not available. Devices were also stratified by interval changes in cylinder length. The 2-year mark was selected for subgroup analysis to allow for sufficient time for tissue expansion to occur and to reduce the influence of early device explantation. Patients who underwent early explantation (e.g., infection, erosion, or oversizing) were likely treated with shorter cylinders due to immediate salvage techniques, development of corporal fibrosis, and need for device downsizing.

Data were tabulated and analyzed in SPSS^®^ (IBM, Armonk, NY, USA). Analyses of categorical and continuous variables were performed using chi-squared test, t-test, and ANOVA analyses. Statistical significance was set at p <0.05.

## RESULTS

During the ten-year study period, 2.749 patients (1.532AMS 700LGX, 717AMS 700CX, and 500 Coloplast Titan) met the inclusion criteria (Table-1). Mean age at the time of first device placement (61 years) was similar between LGX and CX patients (p=0.37); age was not available for Titan patients. Mean time between implants was shorter for LGX (29 months), compared to CX (39 months) and Titan (48 months) patients (p <0.001). Mean initial length of LGX cylinders (19.7 cm) was shorter compared to CX (20.0 cm) and Titan (20.1 cm) (p <0.001) patients.

**Table 1:** Patient and Device Characteristics for AMS 700 LGX, AMS 700 CX, and Coloplast Titan.

		LGX	CX	Titan	p
**Patient Characteristics**					
	Number of patients	717	1532	500	-
	Mean age at first implant, yrs	61	61	-	0.37
	Mean time to replacement, mos	29	39	48	<0.001
**Device Characteristics**					
	Mean initial cylinder length, cm	19.7	20.0	20.1	<0.001
	Mean change in length, cm	0.6	0.5	0.5	0.86
	Mean percent change in length, %	3.3	3.6	3.6	0.77

At the time of device replacement, mean cylinder length (LGX 0.6cm, CX 0.5cm, and Titan 0.5 cm, p=0.86) and percent change of cylinder length (LGX 3.3%, CX 3.6%, and Titan 3.6%, p=0.77) increased equally for all three devices. The Titan increased 0.7 cm, 0.9 cm, 1.0 cm, and 1.3 cm at the time of device replacement 1, 2, 3, and 5 years after the initial placement, respectively. Data for LGX and CX patients was not available for all of those time intervals.

**Figure 1:**
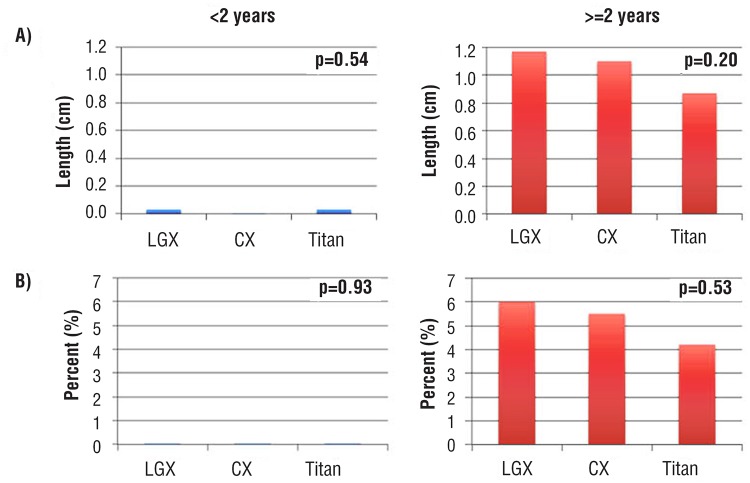
Significant increases in mean cylinder length (A) and mean percent change in cylinder length (B) were seen with device replacement at ≥2 years compared to <2 years for AMS 700LGX, AMS 700CX, and Coloplast Titan (all p <0.001). LGX, CX, and Titan performed similarly within each subcategory (see depicted p values).

At ≥2 years, mean cylinder length (LGX 1.2 cm, CX 1.1 cm, and Titan 0.9 cm), p=0.20) and percent change of cylinder length (LGX 6.5%, CX 6.2%, and Titan 5.5%, p=0.53) increased equally for all three devices (Figure-1). Mean cylinder length did not increase when replaced at <2 years. At ≥2 years, 60% of patients increased >0.5 cm and 40% increased ≥1cm in cylinder length (Table-2). LGX and CX patients both demonstrated increased cylinder length more frequently and in greater magnitude compared to Titan patients (p<0.0001). LGX patients did not demonstrate increased cylinder length compared to CX patients (p=0.12).

**Table 2:** Interval Changes in Cylinder Length for AMS 700 LGX, AMS 700 CX, and Coloplast Titan with Device Replacement at ≥2 Years.

	LGX	CX	Titan
Any decrease in length	33 (10%)	105 (12%)	63 (21%)
0-0.5 cm increase in length	79 (25%)	247 (28%)	55 (18%)
0.5-1 cm increase in length	72 (23%)	196 (22%)	63 (21%)
≥1 cm increase in length	133 (42%)	331 (38%)	119 (40%)

LGX vs. Titan (p<0.0001); CX vs. Titan (p<0.0001); LGX vs CX (p=0.12)

## DISCUSSION

### Tissue Expansion in Surgery

Tissue expansion was first described in 1957 for auricular reconstruction and is most commonly employed today with plastic and breast reconstructive surgery (8). Descriptions of tissue expansion within Urology are limited. Case reports have described good success with expanding penile tissue in children and young adults with scarred skin who required phallic reconstruction in the setting of multiple prior hypospadias and epispadias surgeries (9, 10), Several small studies suggest that an IPP may expand corporal tissue with regular inflation among patients with and without corporal scarring (4, 11). Wilson et al. demonstrated fibrotic corpora secondary to priapism or infection could be stretched with aggressive cycling (11).

We have frequently observed that many patients who undergo IPP replacement are often upsized to larger cylinders, thus prompting our effort to further validate the concept that IPP cylinders may serve as tissue expanders. This unique nationwide, industry-generated database confirmed that 40% and 60% of men undergoing device replacement after two years from initial placement experienced mean increases in device length of ≥1cm and >0.5 cm, respectively.

### Penile Lengthening Procedures and Length Assessment

Evaluating whether an IPP may serve as a tissue expander is clinically important because penile shortening is known to be a frequent source of patient dissatisfaction after IPP insertion (1–6). Patient self-assessment of penile length is notoriously problematic because it tends to be a subjective, emotional, and multifactorial process. One evaluation of over 52,000 men and women identified that although 85% of women were satisfied with their partner's penis size, only 55% of men were satisfied with their own penis size (12). Many concomitant anatomic and technical factors may contribute to the perception of penile shortening after IPP (e.g., prior prostatectomy, Peyronie's disease, priapism, suprapubic fat pad, lack of glans engorgement, inadequate corporal dilation, inappropriate device sizing). In addition, men with refractory erectile dysfunction may suffer from recall bias, since they may not have had any recent, rigid erections. Prior studies evaluating stretched penile length to better characterize length change after IPP placement vary (5, 13). Therefore, without good objective measures of penile length, careful pre-and post-operative counseling becomes even more important to appropriately set patient's expectations.

Numerous ancillary maneuvers (sliding technique, suprapubic lipectomy, suspensory ligament release, autologous fat injections, stretching devices, vacuum protocols, glans injection, ventral phalloplasty) have been developed for penile enlargement, illustrating the importance of penile size among IPP patients (14–16). Complications from these strategies may include penile lumps, nodules, and shaft deformities (7, 17, 18). Our large PIF dataset suggests that aggressive IPP cycling after initial implantation, followed by eventual device upsizing may constitute a treatment strategy for patients with legitimate penile shortening; however, evidence of increased functional length and improved patient satisfaction with device replacement is required before implementing such a treatment strategy.

### Penile Length in IPP Patients

Henry et al. prospectively evaluated penile length and girth measurements for 1 year following Titan IPP placement using an aggressive sizing and cycling protocol. These patients underwent daily inflation for 6 months followed by maximal inflation for 1-2 hours daily for 6-12 months. After 1 year, 65% of patients were pleased with their length, 74% perceived increased length, and most experienced about 1cm increase in stretched penile length with this aggressive cycling regimen. Our nationwide PIF data study similarly identified that many IPP patients who underwent device replacement after two years experienced increased cylinder length. Further evaluation is required to identify the correlation between internal (cylinder) and external (stretched penile and inflated) length.

The selection of which type of prosthesis to use may depend on several characteristics, including patient anatomy, history, and surgeon preference. Because the AMS 700LGX was developed to provide both girth and length expansion, it is commonly recommended for patients with shorter penile lengths. Because the AMS 700CX and Coloplast Titan devices provide only girth expansion, these devices are typically recommended for patients with larger penile lengths. These device features may help to explain why in this study, at the time of first implant, the LGX devices tended to be shorter compared to CX and Titan (p <0.001). Furthermore, LGX devices demonstrated cylinder length increase more frequently and in greater magnitude compared to Titan (p <0.0001), but not CX devices (p=0.12).

### Limitations

Although this 10 years, nationwide dataset study is the largest of its kind to evaluate device length changes over time after IPP implantation, many important limitations exist in this analysis. Conclusions from this study are based on industry obtained PIF data, which lacks important clinical details such as the reason for device explantation or replacement. The reason for device replacement can affect the choice of subsequent device sizing, as men explanted due to infection experienced significant corporal fibrosis and contraction. Furthermore, the method for device sizing is unknown without description of whether some implanters attempted cylinder oversizing during IPP placement or prescribed aggressive postoperative cycling protocols. It is possible that men who inflated their IPP more regularly may have produced more tissue expansion and those who inflated rarely did not.

This nationwide, patient information form data study suggests that increased cylinder length may translate into increased functional length. Further evaluation needs to be conducted to confirm this hypothesis. One counter argument is that implants may compress the flaccid glans and corporal tissue, allowing for increased cylinder length, without increased functional length. Future studies will benefit from patient satisfaction data to evaluate whether increased cylinder length at the time of replacement are of functionally and subjectively beneficial to patient.

## CONCLUSIONS

The IPP does appear to provide some degree of tissue expansion over time, which challenges the common patient perception of penile shortening after IPP insertion. Additional evaluation is needed to evaluate whether increased cylinder length at the time of replacement increases patient satisfaction. Furthermore, the relationship between increased internal corporal length and external penile length remains to be established.

## ABBREVIATIONS

IPP: = inflatable penile prosthesis
PIF: = data: patient information form data
